# Cobalamin Deficiency May Induce Astrosenescence—An In Vitro Study

**DOI:** 10.3390/cells11213408

**Published:** 2022-10-28

**Authors:** Zuzanna Rzepka, Jakub Rok, Justyna Kowalska, Klaudia Banach, Dorota Wrześniok

**Affiliations:** Department of Pharmaceutical Chemistry, Faculty of Pharmaceutical Sciences in Sosnowiec, Medical University of Silesia in Katowice, 4 Jagiellońska Str., 41-200 Sosnowiec, Poland

**Keywords:** astrosenescence, cobalamin deficiency, vitamin B12, cellular senescence

## Abstract

Cobalamin (vitamin B12) deficiency is one of the major factors causing degenerative changes in the nervous system and, thus, various neurological and psychiatric symptoms. The underlying cellular mechanism of this phenomenon is not yet fully understood. An accumulation of senescent astrocytes has been shown to contribute to a wide range of pathologies of the nervous system, including neurodegenerative disorders. This study aimed to investigate whether cobalamin deficiency triggers astrosenescence. After inducing cobalamin deficiency in normal human astrocytes in vitro, we examined biomarkers of cellular senescence: SA-β-gal, p16^INK4A^, and p21^Waf1/Cip1^ and performed cell nuclei measurements. The obtained results may contribute to an increase in the knowledge of the cellular effects of cobalamin deficiency in the context of astrocytes. In addition, the presented data suggest a potential causative agent of astrosenescence that has not been proven to date.

## 1. Introduction

Cobalamin, also known as vitamin B12, is crucial for cell functioning, particularly DNA synthesis, methylation, and mitochondrial metabolism. A subclinical deficiency of this vitamin could affect approx. 26% of the general population. Risk factors of cobalamin deficiency include old age, gastric or small intestine resections, inflammatory bowel disease, and veganism, as well as the use of metformin, proton pump inhibitors or histamine H2 blockers. Vitamin B12 deficiency can present with non-specific clinical features and, in severe cases, with hematological or neurological abnormalities [1,2,3]. The most common neurological symptoms of cobalamin deficiency are myelopathy, neuropathy, dementia, disturbance of position sense, and spastic paraparesis or tetraparesis [4]. The cellular mechanisms of these abnormalities have not yet been fully explained. Our previous studies [5,6] have shown that vitamin B12 deficiency significantly affects the homeostasis of human astrocytes, which may underlie neuropsychiatric disorders associated with hypocobalaminemia.

Cellular senescence is a form of irreversible growth arrest. There are two main categories: replicative senescence (which occurs secondary to telomere shortening) and stress-induced premature senescence [7,8]. The latter has since been identified as a response to numerous stressors, including hypoxia, chemotherapeutic drugs, ionizing radiation, oncogene activation, oxidative stress and mitochondrial dysfunction. Cellular senescence regulates physiological and homeostatic processes, particularly during embryonic development and wound healing, but can also be a pathological process that contributes to ageing and various diseases [7,8,9]. The most widely used biomarker for cellular senescence is senescence-associated β-galactosidase (SA-β-gal), which is defined as β-galactosidase activity detectable at pH 6.0. It has been shown that β-galactosidase activity is significantly higher in senescent cells than in pre-senescent cells. Senescent cells are also characterized by enlarged and flat cell morphology, increased p16^INK4A^ and p21^Waf1/Cip1^ protein expression [8,9], and an expanded nuclear area [10]. Moreover, some senescent cells could show an altered reaction to apoptotic stimuli [11]; however, this is not a general feature of senescent cells and the interplay between senescence and apoptosis is cell-type-specific [12,13].

Astrocytes are a class of glial cells that sustain homeostasis of the central nervous system (CNS). These cells provide structural and metabolic support to neurons, control blood–brain barrier and constitute the main defensive system of the CNS. Recent studies have demonstrated that defects in astrocyte function are related to a wide range of neuropathologies [14].

Astrocytes have been shown to undergo replicative senescence and premature senescence. This phenomenon is termed astrosenescence. In vivo studies have revealed that an accumulation of senescent astrocytes could have implications for the neurological disorders, such as Alzheimer disease and Huntington disease, and for the aging brain [14,15]. Nevertheless, the role of astrosenescence in CNS pathologies, as well as the factors that can provoke this condition, are not fully understood and require further research.

The aim of the current study was to investigate whether cobalamin deficiency is an astrosenescence-inducer. For this purpose, we used a previously developed hypocobalaminemia model based on the culture of normal human astrocytes (NHA) in the presence of the cobalamin antagonist, hydroxycobalamin [*c*-lactam]. After inducing cobalamin deficiency in NHA, we examined biomarkers of cellular senescence: SA-β-gal, p16^INK4A^, and p21^Waf1/Cip1^ and measured cell nuclei. In addition, we examined whether the cobalamin deficiency state affects the sensitivity of cultured astrocytes to apoptosis. We believe that the obtained results will contribute to an increase in knowledge about the cellular effects of cobalamin deficiency in the context of astrocytes. In addition, the presented data suggest a potential causative agent of astrosenescence that has not been proven to date.

## 2. Materials and Methods

### 2.1. Cell Culture

Gibco Human Astrocytes (Thermo Fisher Scientific; Waltham, MA, USA) were cultured in Gibco Astrocyte Medium supplemented with antibiotics, as described previously [5].

### 2.2. The Induction of Cobalamin Deficiency

In the current study, we applied a previously developed [5] experimental model of vitamin B12 deficiency in normal human astrocytes. In brief, the NHA were seeded (100,000 cells per T-25 flask) and cultured for 27 days with 20 µg/mL of hydroxycobalamin [*c*-lactam] (HCCL), which is a cobalamin antagonist. HCCL was synthesized by Prof. Dorota Gryko from Institute of Organic Chemistry, Polish Academy of Science. according to the previously described method [16].

### 2.3. Analysis of Senescence-Associated β-Galactosidase Activity

The CellEvent Senescence Green Detection Kit (Thermo Fisher Scientific; Waltham, MA, USA) was used. The fluorescein-based probe contains two galactoside moieties, making it a target for cellular β-galactosidase. The enzymatically cleaved product emits fluorescence (absorption/emission maxima = 490/514 nm). In brief, the cells were grown on Nunc Lab-Tek 4-well Chambered Coverglass (Thermo Fisher Scientific; Waltham, MA, USA). After washing with PBS, the cells were fixed with 2% paraformaldehyde and then washed with 1% bovine serum albumin (BSA). Next, the cells were incubated with the diluted CellEvent Senescence Green Probe for 2 h and then washed 3 times with PBS. The samples were observed using a Nikon A1R Si confocal system (Nikon Instruments, Amsterdam, The Netherlands). Nikon NIS Elements AR 4.51 software was applied for image analysis.

### 2.4. Western Blot Analysis

The analysis was performed according to the protocol presented in our previous article [6]. In this study, we used the following primary antibodies (Abs): anti-p16^INK4A^ rabbit Ab (1:1000 dilution); anti-p21^Waf1/Cip1^ rabbit Ab (1:1000 dilution); anti-GAPDH rabbit Ab (1:1000 dilution), all purchased from Cell Signaling (Danvers, MA, USA).

### 2.5. Confocal Imaging and Analysis

The method used was described earlier [6]. Primary Abs obtained from Cell Signaling (Danvers, MA, USA) was used in the following dilutions: anti-p16^INK4A^ rabbit Ab (1:800 dilution), anti-p21^Waf1/Cip1^ rabbit antibody (1:400 dilution).

### 2.6. Mitochondrial Membrane Potential Assay

The JC-1 staining probe and image cytometer NucleoCounter NC-3000 (ChemoMetec; Lillerød, Denmark) was applied to determine the mitochondrial membrane potential (Δψm). The procedure was previously decribed in our paper [17].

### 2.7. Annexin V Assay

The annexin V assay and image cytometer NucleoCounter NC-3000 were applied to analyze the process of cell apoptosis. The protocol was described previously [17].

### 2.8. Statistical Analysis

Statistical analysis obtained data was performed using GraphPad Prism 8.0.1 software (GraphPad Software; San Diego, CA, USA). In all experiments mean values ± standard deviation (SD) of at least three separate experiments. Differences between groups were assessed by the unpaired *t*-test (in the case of normally distributed data) or the Mann–Whitney U test (for data following non-normal distribution), as indicated in the figure captions. A *p*-value < 0.05 was used as the cutoff for statistical significance.

## 3. Results

### 3.1. The Detection of SA-β-Gal in Cobalamin-Deficient Astrocyte

**Figure 1:**
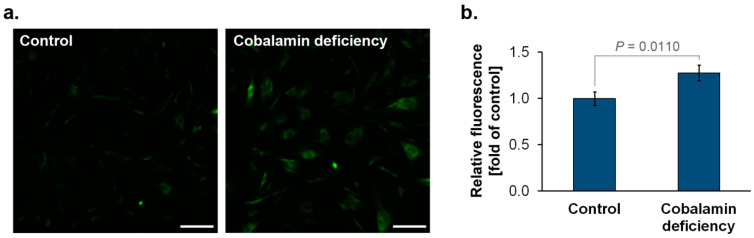
Cobalamin deficiency caused an increase in SA-β-gal activity in normal human astrocytes. The cells were stained with the CellEvent Senescence Green probe. A fluorescence signal from the enzyme-cleaved product was analyzed using a confocal system. Representative microscopic images (**a**) and a bar graph (**b**) corresponding to the mean ± SD of three independent experiments were presented. *p* value from the unpaired *t*-test test is indicated. The scale bar shows 200 µm.

SA-β-gal is an enzyme that accumulates in the lysosomes of senescent cells, where it hydrolyses β-galactoside [18]. We analyzed SA-β-gal activity in astrocytes from experimental model of hypocobalaminemia. As presented in Figure 1, in comparison to the control, vitamin B12-deficient astrocytes showed increased SA-β-gal activity, which may be considered a biomarker of cellular senescence.

### 3.2. The Assessment of p16^INK4A^ and p21^Waf1/Cip1^ Expression in Astrocytes upon Cobalamin Deficiency

**Figure 2:**
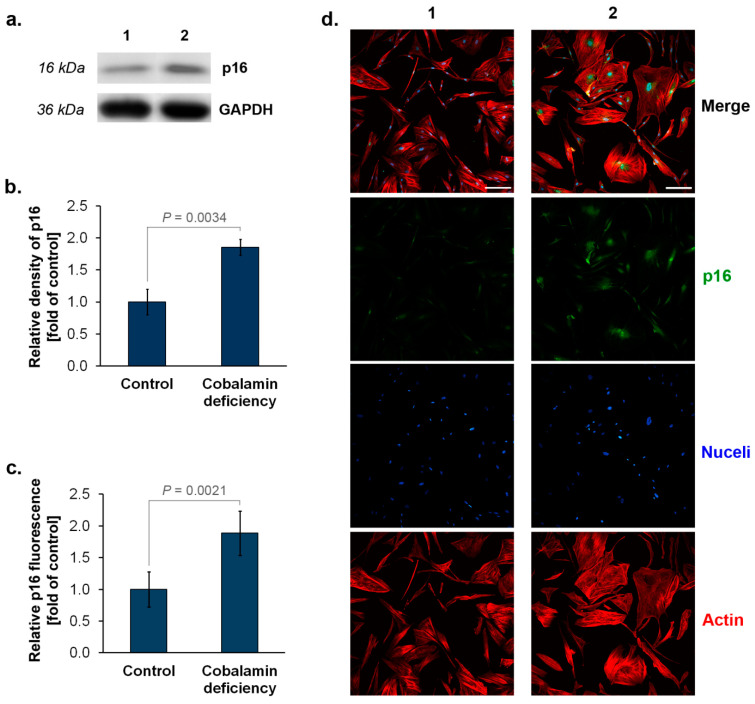
Elevated expression of p16 (p16^INK4A^) in cobalamin deficient astrocytes. Representative blot (**a**) and quantification of Western blot bands by densitometry, where 1 = Control, 2 = Cobalamin deficiency (**b**). Data from fluorescence intensity measurements (**c**) and representative microphotographs of immunolabeled p16 protein (**d**). Bar graphs represent the mean ± SD of three independent experiments. *p* values from the unpaired *t*-test test are indicated. The scale bar shows 200 µm.

**Figure 3:**
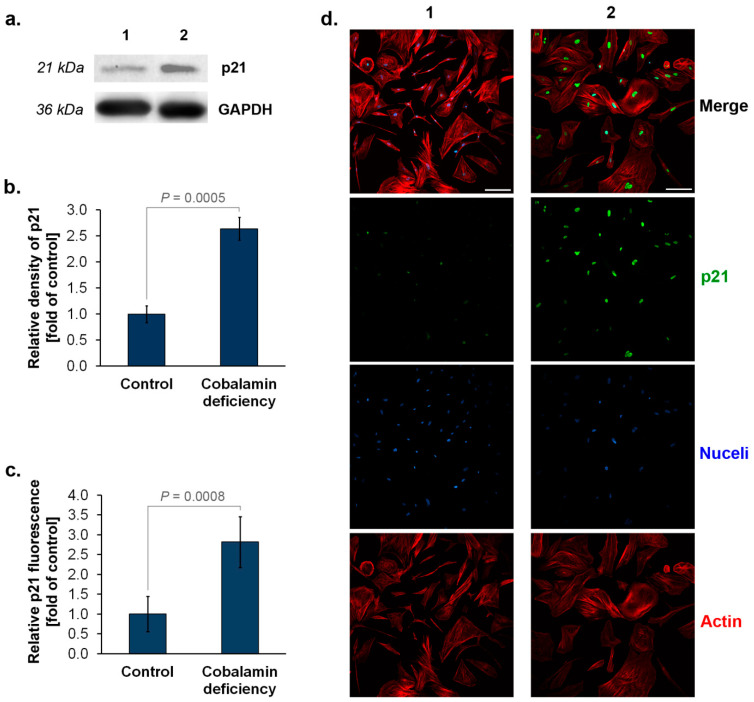
Elevated expression of p21 (p21^Waf1/Cip1^) in cobalamin deficient astrocytes. Representative blot (**a**) and quantification of Western blot bands by densitometry, where 1 = Control, 2 = Cobalamin deficiency (**b**). Data from fluorescence intensity measurements (**c**) and representative microphotographs of immunolabeled p21 protein (**d**). Bar graphs represent the mean ± SD of three independent experiments. *p* values from the unpaired *t*-test test are indicated. The scale bar shows 200 µm.

High levels of cyclin-dependent kinase inhibitors p16^INK4A^ and p21^Waf1/Cip1^ may indicate senescent cells [8,9,19]. We assessed the expression of these proteins in vitamin B12 deficient- and untreated (control) astrocytes. Western blot analysis showed that cobalamin deficiency in human astrocytes results in about two times higher levels of p16^INK4A^ protein compared to the control (Figure 2a,b). Similarly, analysis based on immunofluorescence imaging, in addition to visualizing the protein in the studied cells, provided data indicating a significant increase in protein expression in astrocytes under vitamin B12 deficit (Figure 2c,d). In the case of p21^Waf1/Cip1^, about a 2.5-fold increase in the protein level in cobalamin-deficient cells was estimated by both Western blot (Figure 3a,b) and immunofluorescence (Figure 3c,d) techniques.

### 3.3. The Evaluation of Cell Nuclei Area in Cobalamin Deficient Astrocytes

**Figure 4:**
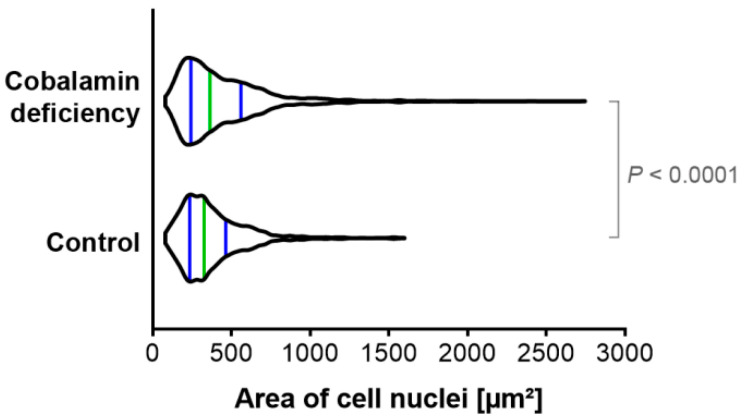
Cobalamin deficiency in astrocytes affects cell nuclei area. The cell nuclei were stained with SYTO Deep Red Nucleic Acid Stain and then visualized using a confocal system. Image analysis included creating the binary layer through thresholding in the channel of SYTO Deep Red Stain in the Nikon NIS Elements AR software. Then, the regions of interest (ROIs) were created, and an automated measurements tool was used to determine the area of the individual cell nuclei within each of the analyzed microphotographs. The violin plot depicts data distribution with a green line indicating the median, and blue lines indicating the first and the third quartiles. *p* value from the Mann–Whitney U test is indicated.

Nuclear area has been demonstrated to expand during senescence [10]. Our quantitative analysis has indicated that the population of cobalamin-deficient astrocytes differs from the control culture in terms of cell nuclei area, as presented in Figure 4. We have revealed that vitamin B12 deficiency promotes the occurrence of astrocytes with enlarged nuclei, which may indicate cellular senescence.

### 3.4. The Assessment of Apoptosis in the Astrocyte-Based Model of Hypocobalaminemia

**Figure 5:**
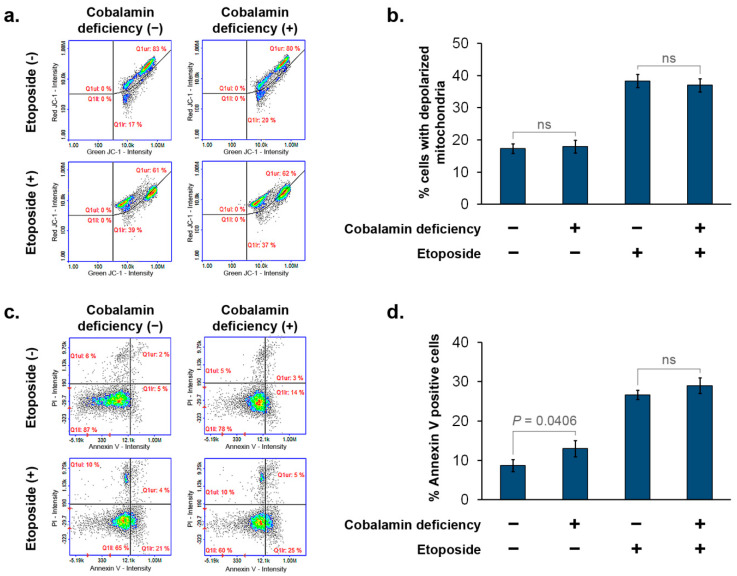
Cobalamin deficiency in astrocytes does not affect etoposide-dependent induction of apoptosis. In order to induce apoptosis, the cells were treated with 300 µM etoposide for 48 h. Then, mitochondrial membrane potential assay (**a**,**b**) and annexin V staining (**c**,**d**) were performed using image cytometry. Representative scatterplots and bar graphs (corresponding to the mean ± SD of three independent experiments were presented. *p* value from the unpaired *t*-test test is indicated).

We investigated the effect of cobalamin deficiency on cell susceptibility to apoptosis by the use of image cytometry. Mitochondrial potential (Δψm) analysis and annexin V assay were performed. Four populations of astrocytes were analyzed: (i) NHA cultured in a growth medium; (ii) NHA cultured in a growth medium with the addition of HCCL (20 µg/mL) for 27 days (cobalamin-deficient cells); (iii) NHA cultured in a growth medium and then for 48 h with 300 µM etoposide; (iv) NHA cultured in a growth medium with the addition of HCCL (20 µg/mL) for 27 days, and then for 48 h with 300 µM etoposide. The treatment of astrocytes with etoposide resulted in the depolarization of mitochondria with a decrease in Δψm (Figure 5a,b). A slight increase in the percentage of Annexin V-positive cells was also observed after the cell treatment with etoposide (Figure 5c,d). Importantly, based on both apoptosis assays used in this study, no differences were shown between astrocytes treated only with etoposide (cobalamin deficiency −, etoposide +) and cobalamin-deficient astrocytes treated with etoposide (cobalamin deficiency +, etoposide +). Thus, it may be concluded that human astrocytes in a state of cobalamin deficiency are neither more nor less resistant to the pro-apoptotic agent than the control cells. However, this issue should be further studied with other apoptotic stimuli.

## 4. Discussion

Neurological disorders are the main cause of disability and rank among the predominant causes of death worldwide [20]. The elucidation of their pathophysiological mechanisms is one of the greatest scientific challenges today. Many studies point to the huge role that astrocyte dysfunctions play in multiple sclerosis, Alzheimer disease (AD), Parkinson disease (PD), Huntington disease and other neuropsychiatric disorders [21,22,23,24].

Cobalamin deficiency is one of the major factors causing degenerative changes in the nervous system, and thus neurological symptoms such as symmetric dysesthesia, disturbance of position sense, spastic paraparesis or tetraparesis and dementia [4,25]. Existing studies show a link between B12 hypovitaminosis and leading neurodegenerative diseases, such as AD, PD and depression [26,27,28,29]. Moreover, cobalamin deficiency has been negatively correlated with cognitive functioning in healthy elderly subjects [4,30]. The underlying mechanism of neuropsychiatric dysfunctions in patients with cobalamin deficiency has not yet been fully understood.

Previously we developed an experimental model of cobalamin deficiency in normal human astrocytes [5]. The model was then used for further analysis, in which we indicated that astrocytes, upon cobalamin deficiency, may undergo a homeostasis imbalance including inhibited cell proliferation, cell cycle arrest in G2/M phase, activation of caspase 3/7, 8 and 9, cell hypertrophy, and GFAP and vimentin overexpression [5,6]. Zhang et al. [31] revealed the decrease in brain vitamin B12 status across the lifespan, which suggests the role of cobalamin deficiency in brain aging.

Recent studies suggest that astrosenescence has a severe impact on neurodegenerative disorders and brain aging [15,32]. Bhat et al. [33] demonstrated a significant increase in senescent (p16^INK4A^-positive) astrocytes in the frontal cortex of AD patients compared with non-AD adult control subjects of similar ages. Vazquez-Villaseñor et al. [34] observed the significantly higher percentage of p16^INK4A^-positive and p21^Waf1/Cip1^-positive cells in the frontal cortex of patients with motor neuron disease, which suggest the involvement of senescence in the progression of this disorder. Recently, it has been proposed that targeting senescent cells by senolytics can ameliorate the effect of brain aging, thus preserving cognitive function into old age [32]. Senolytic agents are recognized as a promising group of therapeutics and are widely studied [35].

The aim of the current study was to determine whether a senescence activation occurs in astrocytes under cobalamin deficiency. We induced cobalamin deficiency in the culture of normal human astrocytes and then examined key markers of cellular senescence.

Our analysis indicated that vitamin B12-deficient astrocytes show increased SA-β-gal, a well-recognized biomarker of senescence. Moreover, we found that cobalamin deficiency in the studied cells may resulted in an overexpression of p16^INK4A^ and p21^Waf1/Cip1^. The disturbance of DNA synthesis due to cobalamin deficiency may probably induce p21 expression, as demonstrated by Kwan et al. [36] for clots of megaloblastic anemia patients. Nevertheless, the role of the two cyclin-dependent kinase inhibitors, p16^INK4A^ and p21^Waf1/Cip1^, in cellular senescence has been widely investigated. p21^Waf1/Cip1^ blocks cyclin-dependent kinase 2 (CDK2) activity, leading to cell-cycle arrest and the initiation of senescence. Persistent cellular stress leads to the upregulation of p16^INK4A^, triggering the p16^INK4A^/RB pathway and irreversible cellular arrest [37]. The expression of p16^INK4A^ protein is highly dynamic, being largely undetectable in healthy young tissues, but rising in many tissues with aging or after injury [38]. Bitto et al. [39] indicated that after oxidative stress or proteasome inhibition human and mouse astrocytes undergo senescence exhibiting SA-β-gal activity. They revealed that an increase in p21^Waf1/Cip1^ protein level in senescent astrocytes is accompanied by the overexpression of p16^INK4A^. Interestingly, according to the study by Bitto et al. [39], astrocytes were more sensitive to stress-induced senescence than fibroblasts. These results suggest that the central nervous system may be particularly vulnerable to premature senescence.

Recently, Heckenbach et al. [10] examined the morphology of cell nuclei in senescent dermal fibroblasts. They provided strong evidence that there is a significant difference in nuclear area between non-senescent and senescent cells. Similarly, in this paper, we have indicated that the population of cobalamin-deficient astrocytes, in addition to well-recognized senescence biomarkers, may exert the cell nuclei enlargement, which may be a consequence of cell-cycle arrest in the G2/M phase.

The state of senescence may affect the susceptibility of cells to apoptosis; however, this interplay has been shown to be cell-type-specific [13]. In the current study, we indicated that exposure to etoposide induced apoptosis to the same extent in senescent and non-senescent astrocytes. Similarly, in their in vitro study, Seluanov et al. [40] demonstrated that the senescent phenotype of human fibroblasts did not affect their response to etoposide, which was found to be an inducer of p53-independent apoptosis. In contrast, senescent fibroblasts were resistant to p53-dependent apoptotic agents such as actinomycin D, low-dose cisplatin or UVB irradiation. Thus, the authors have concluded that apoptosis resistance in senescent fibroblasts may result from an alteration in intracellular p53 signaling. The resistance to undergoing programmed cell death in the case of fibroblasts, was also observed by other researchers [41,42]. Conversely, senescent porcine pulmonary artery endothelial cells [43] and senescent human umbilical vein endothelial cells [41] were shown to be more susceptible to apoptosis than the non-senescent counterparts. Considering the examples and results presented in this paper, it can be hypothesized that senescent cells present a complex and diverse phenotype, which varies substantially by cell type and the causative agent.

To the best of our knowledge, this is the first study to investigate whether cobalamin deficiency induces astrosenescence. Our research sheds light on the cellular mechanism of nervous system disorders associated with hypocobalaminemia, pointing to the potential role of senescent astrocytes. We believe that the findings could form the basis for further research into the potential efficacy of senolytics in the treatment of clinical implications of cobalamin deficiency.

## Data Availability

Not applicable.
